# Association between endothelin-1 and diabetic retinopathy: a systematic review and meta-analysis

**DOI:** 10.3389/fendo.2026.1754896

**Published:** 2026-03-02

**Authors:** Xin Sun, Yan An, Xiandong Zeng, Yanhua Jiang

**Affiliations:** 1 Department of Endocrinology, First Affiliated Hospital of Soochow University, Suzhou, China; 2 Department of Ophthalmology, Fourth People`s Hospital of Shenyang, Shenyang, China

**Keywords:** diabetic retinopathy, DR, endothelin-1, ET-1, meta-analysis

## Abstract

**Background:**

Diabetic retinopathy (DR) is a common chronic complication of diabetes mellitus. Endothelin-1 (ET-1) has been identified as a key regulator of various ocular functions, including vascular perfusion, aqueous humor dynamics, and retinal ganglion cell survival. Substantial evidence further underscores the critical involvement of ET-1 in the pathogenesis and progression of DR. Elevated ET-1 levels have been reported in patients with DR; however, findings across studies are inconsistent.

**Aim:**

This meta-analysis aimed to statistically evaluate the level of ET-1 in patients with DR.

**Methods:**

A systematic literature search was conducted across five electronic databases (PubMed, Web of Science, OVID, Elsevier Science Direct, and Wiley Online Library). The search strategy targeted the terms “Endothelin-1” or “ET-1” in conjunction with “Diabetic retinopathy” or “DR” in title and abstract fields. Results are presented as standardized mean differences (SMD) with 95% confidence intervals (CI).

**Results:**

Ten articles (346 cases and 425 controls) were included in the meta-analysis. The results of the meta-analysis indicated that the circulating ET-1 in patients with DR was significantly higher than that of the controls (SMD: 1.73, 95% CI: 0.90, 2.56). Furthermore, circulating ET-1 in patients with DR was also significantly higher than those in healthy individuals or diabetic patients without retinopathy, respectively.

**Conclusions:**

This meta-analysis is the first to systematically assess ET-1 levels in patients with DR. The findings of this study indicate the potential application of ET-1 as a biomarker for monitoring DR progression.

**Systematic Review Registration:**

https://www.crd.york.ac.uk/prospero/, identifier CRD420251156225.

## Introduction

Diabetic retinopathy (DR) is a common chronic complication of diabetes mellitus (DM) and a leading cause of acquired blindness among the working-age population. Its prevalence is positively correlated with the duration of DM, with incidence rates reaching approximately 50% in patients with a disease duration of about 10 years and 80% in those with a disease duration of over 15 years (1). In developed countries, DR accounts for more than 10% of all blindness cases. Furthermore, recent epidemiological studies have indicated that over one-third of patients with diabetes worldwide currently present with retinopathy or varying degrees of diabetes-related ocular damage (2). The fundamental pathophysiological processes of DR include retinal capillary endothelial injury, dysregulation of the capillary network, neurodegeneration, loss of retinal pericytes, and impairment of the blood-retinal barrier. These pathological changes contribute to capillary leakage and capillary occlusion, leading to vision loss (3, 4).

Extensive research conducted over the years has indicated that DR likely results from the combined effects of multiple factors and pathways. These pathways include the abnormal activation of the polyol pathway, the excessive activation of the hexosamine pathway, oxidative stress, the enhanced activity of the renin–angiotensin system, the accumulation of advanced glycation end products, and persistent low-grade inflammation (5, 6). Currently, several treatments are available for DR, such as anti-Vascular Endothelial Growth Factor (VEGF) agents, corticosteroid injections, and laser therapy. However, these approaches are mostly invasive and primarily target late-stage disease, making them insufficient to meet the needs of the growing patient population. Recently, research has been shifting toward safer, non-invasive strategies aimed at simultaneously modulating multiple key pathways involved in DR progression, including oxidative stress, inflammation, apoptosis, and pathological angiogenesis (7, 8). Nevertheless, more effective biomarkers and therapeutic targets are still needed (9).

In 1988, endothelin-1 (ET-1) was first isolated and purified from cultured porcine aortic endothelial cells by Yanagisawa et al. (10). Subsequently, ET-1 was identified as a key regulator of various ocular functions, including vascular perfusion, aqueous humor dynamics, and retinal ganglion cell survival. ET-1 induces vasoconstriction, exacerbating retinal ischemia; increases vascular permeability, leading to blood-retinal barrier disruption; promotes the proliferation of vascular cells, contributing to neovascularization; and drives inflammatory responses (11). Morise T et al. reported a significant increase in ET-1 levels of patients with DR than the controls (12). However, the results of other similar studies were inconsistent with those of earlier investigation. Adamiec-Mroczek J et al. did not observe a statistically significant increase in ET-1 levels in DR patients versus controls (13). Notably, Ugurlu N et al. reported that ET-1 concentrations in DR patients were actually lower than those in control subjects (14).While, there are some differences in the enrolled populations and study methodologies across the various studies. Therefore, the relationship between ET-1 levels and DR remains controversial. In elucidating this controversial association, a meta-analysis was conducted to quantitatively assess the level of ET-1 in patients with DR.

## Methods

### Search

Comprehensive searches were conducted in the following electronic databases: PubMed, Web ofScience, OVID, Elsevier Science Direct, and Wiley Online Library. The search strategy included the terms “Endothelin-1” or “ET-1” combined with either “Diabetic Retinopathy” or “DR” in the title or abstract. All relevant publications from 1980 through 2025 were considered. In addition, reference lists of the retrieved articles were reviewed to identify other potentially eligible studies; however, unpublished reports were excluded. A completed Preferred Reporting Items for Systematic Reviews and Meta-Analyses (PRISMA) checklist is available in the Supplementary Data (**Supplementary Table S1**). The protocol for this systematic review and meta-analysis was registered with PROSPERO (registration number: CRD420251156225).

### Inclusion criteria

Studies were included in the meta-analysis if they met the following criteria: (1) the studies used a case–control or cohort design; (2) the studies reported detailed measurements of circulating ET-1 levels in patients with DR and control groups; (3) the studies were published in English.

### Exclusion criteria

Studies were excluded based on the following criteria: unavailability of the full text, duplication of publications, incomplete or non-convertible data, and the implementation of interventions in either the experimental or control groups that did not comply with the study protocol. Furthermore, exclusion applied to studies with significant methodological flaws, as well as non-human research, review articles, conference abstracts, case reports, and editorial commentaries.

### Data extraction and risk of bias

Two investigators independently performed literature screening, data extraction, and cross-validation. Any discrepancies were resolved through discussion or by consulting a third reviewer. Screening began with a review of article titles, followed by the exclusion of irrelevant studies. Subsequently, abstracts and full texts of the remaining articles were examined to assess eligibility for inclusion. When necessary, corresponding authors were contacted via email to obtain missing data. The extracted information included the title, first author, year of publication, study location, sample size, participant age per group, and relevant outcome indicators.

The Newcastle–Ottawa Scale (NOS), which was recommended by the Cochrane Collaboration for assessing the quality of observational studies, was used to evaluate risk of bias (15, 16). Two researchers independently rated each study, compared scores, and resolved inconsistencies through consensus. In cases where agreement could not be reached, a third researcher was consulted to make the final determination. The NOS tool comprises three domains and eight items, with total scores ranging up to 9.

### Statistical analysis

The results of this meta-analysis are expressed as standardized mean differences (SMDs) with 95% confidence intervals (CIs). Between-study heterogeneity was evaluated using Cochran’s Q test and the *I*² statistic. An *I*² value below 50% indicated low-to-moderate heterogeneity, in which case a fixed-effect model was applied; otherwise, a random-effect model was used. Sensitivity analysis was conducted to examine the impact of individual studies on the overall results. Potential publication bias was assessed using Begg’s and Egger’s tests, where a *P*-value of <0.05 was considered statistically significant. All analyses were performed using Stata version 12.0 (StataCorp, College Station, TX, USA).

## Results

**Figure 1:**
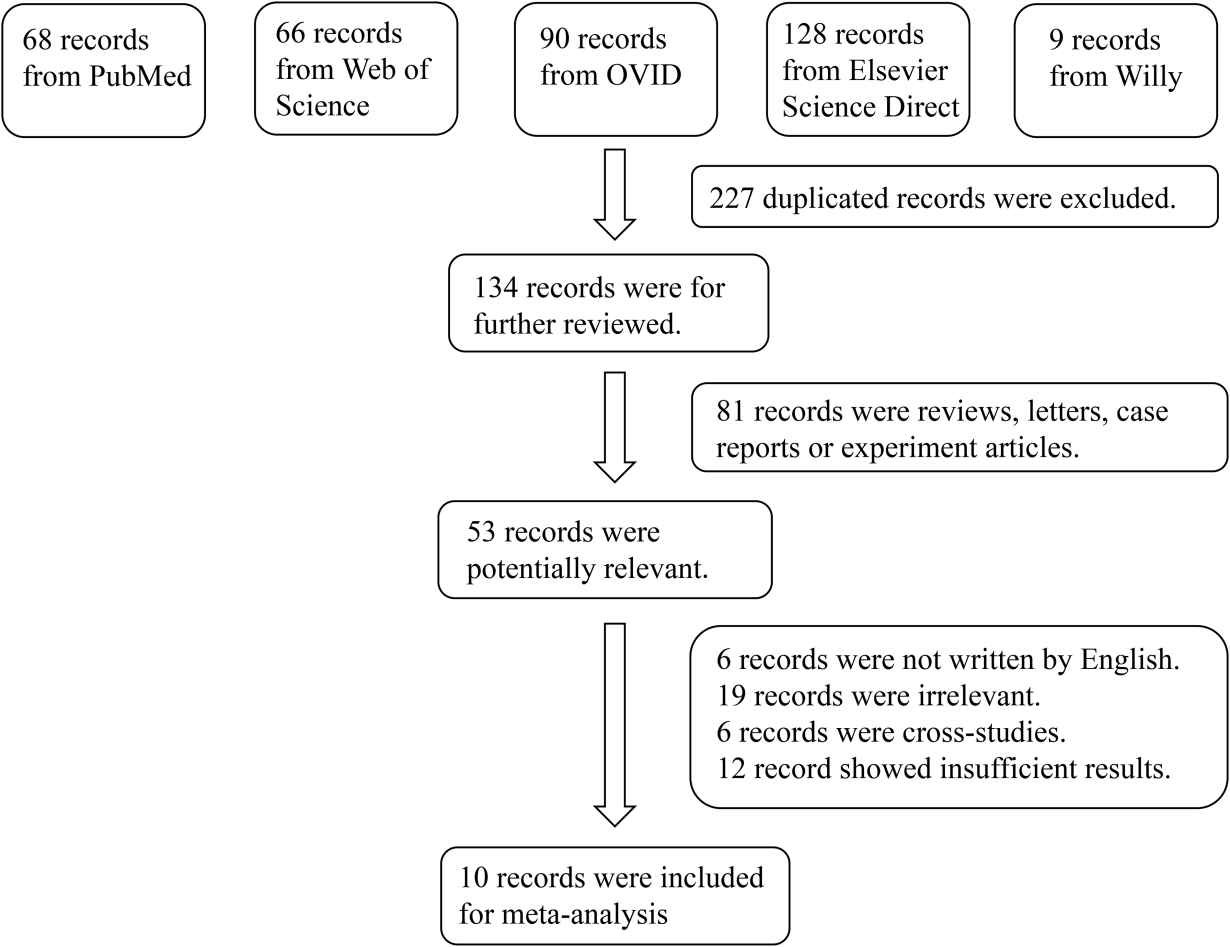
Flowchart of the detailed procedure for the inclusion or exclusion of selected studies.

The initial literature search identified 361 relevant publications from the five electronic databases. Although manual screening of reference lists was conducted, no additional studies met the inclusion criteria. After carefully screening, 10 articles comprising a total of 346 cases and 425 controls were included in the final meta-analysis (12–14, 17–22). The flowchart of the study selection process is shown in **Figure 1**, and the key characteristics of the included studies are presented in **Table 1**.

**Table 1:** Study characteristics of the published studies included in the meta-analysis.

Author	Publication year	Region	Number (n)		Level of endothelin-1		Sex (M/F)		Age (years)		NOS
			Case	Control	Case	Control	Case	Control	Case	Control	
Morise T (12)	1995	Japan	10	22	1.01 ± 0.07 pmol/L	0.55 ± 0.07pmol/L	5/5	11/11	44 ± 4	43 ± 4	6
Best RM (17)	1999	UK	7	14	13.5 ± 2.1 pg/ml	9.49 ± 6.29pg/ml	0/7	0/14	30.5	32.2	5
Roldan-Pallares M (18)	2007	Spain	25	50	3.49 ± 0.25 pg/ml	2.43 ± 0.26 pg/ml	15/10	25/25	60.04 ± 1.63	59.20 ± 1.79	7
Zhu H (19)	2007	China	96	144	195.2 ± 50.37 pg/ml	156 ± 26.5 pg/ml	46/50	75/69	63.04 ± 10.69	57.16 ± 10.98	7
Adamiec-Mroczek J (13)	2010	Poland	19	15	1.01 ± 1.47 fmol/mL	0.45 ± 0.23 fmol/mL	7/12	6/9	64.63 ± 8.38	63.00 ± 14.58	6
Mohamed TA (20)	2010	Egypt	40	30	20.3 ± 5.3 ng/l	2.3 ± 1.0 ng/l	17/23	Sex-matched	48.5 ± 12.2	Age-matched	6
Ugurlu N (14)	2013	Turkey	30	59	8.91 ± 4.69 fmol/mL	9.64 ± 4.76 fmol/mL	–	–	31.83 ± 2.38	31.71 ± 0.73	5
Vingolo EM (21)	2017	Italy	9	11	0.38 ± 0.13 ng/ml	0.43 ± 0.07 ng/ml	4/5	6/3	63.33 ± 12.52	67.18 ± 9.66	6
Niranjan G (22)	2019	India	60	30	15.58 ± 3.96 pg/ml	11.5 ± 2.1 pg/ml	–	–	56 ± 7.8	54 ± 8.5	7
Xu K (23)	2020	China	50	50	26.9 ± 1.3 ng/l	25.4 ± 1.7 ng/l	29/21	25/25	49 ± 16	48 ± 15	6

NOS, Newcastle-Ottawa Scale.

### Results of the meta-analysis

**Figure 2:**
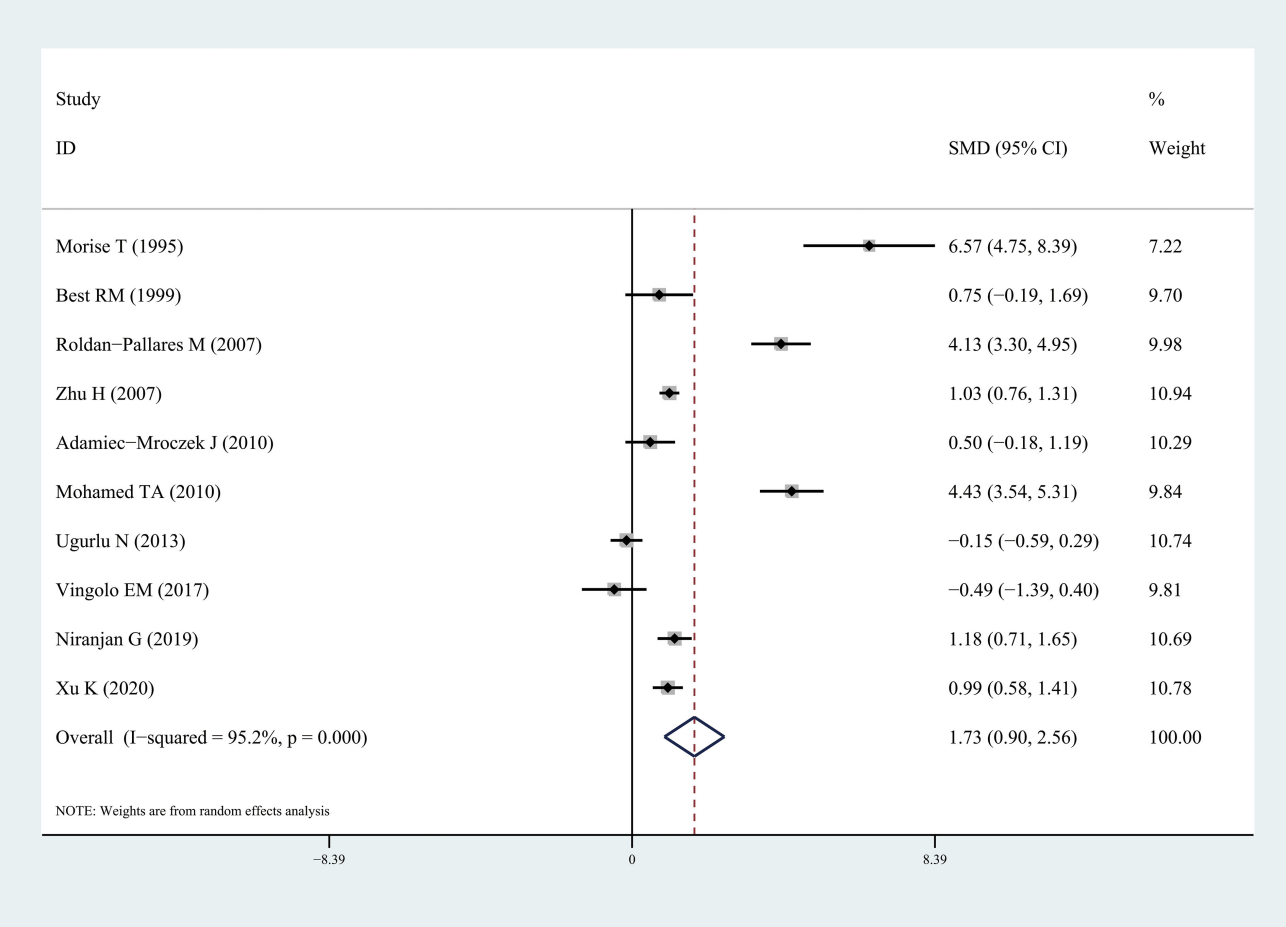
Forest plots of circulating endothelin-1 in patients with diabetic retinopathy compared to the control. Diamond represents the pooled SMDs at 95% CI. SMD, standardized mean difference; CI, confidence interval.

**Figure 3:**
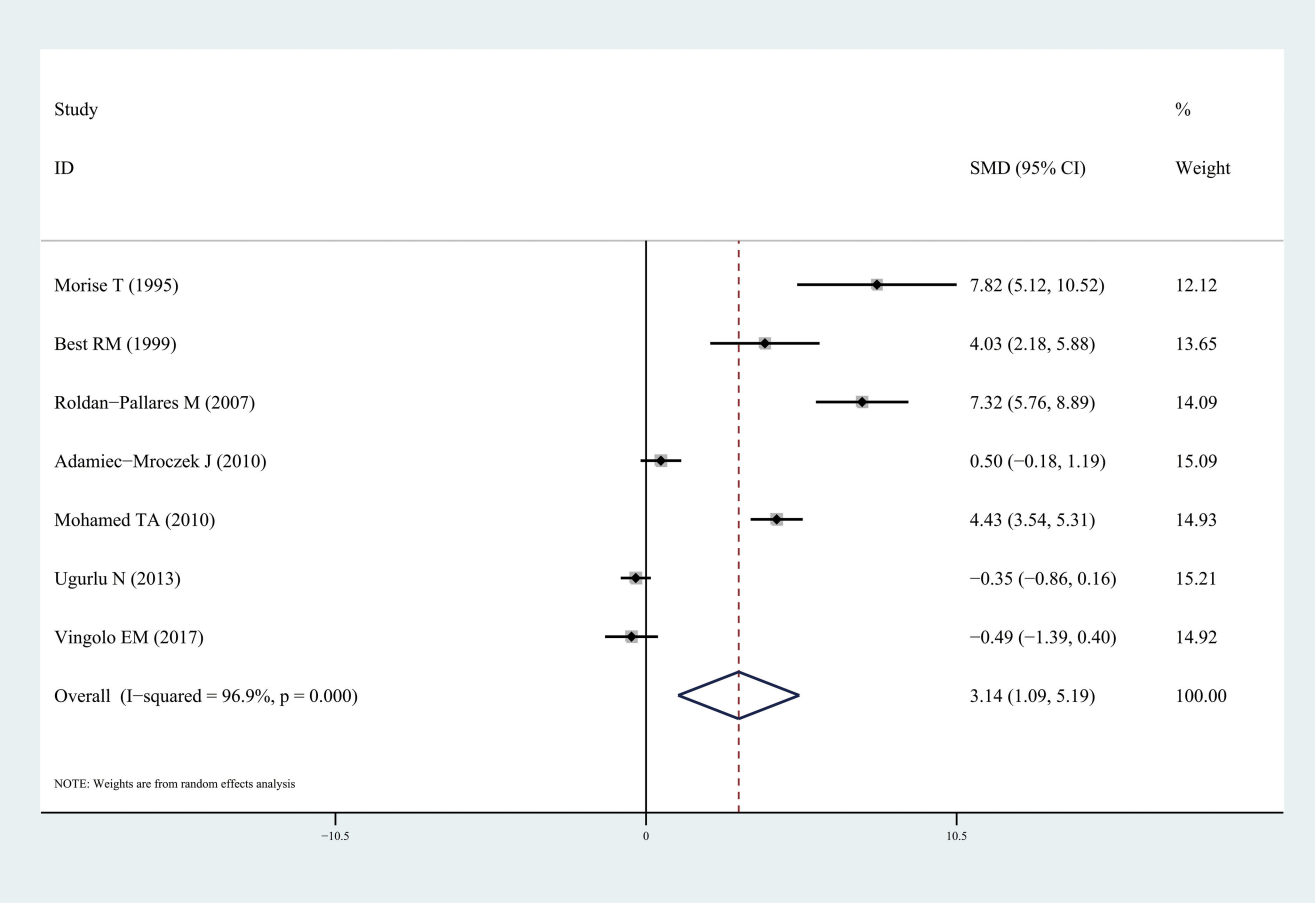
Forest plots of circulating endothelin-1 in patients with diabetic retinopathy compared to the healthy individuals. Diamond represents the pooled SMDs at 95% CI. SMD, standardized mean difference; CI, confidence interval.

**Figure 4:**
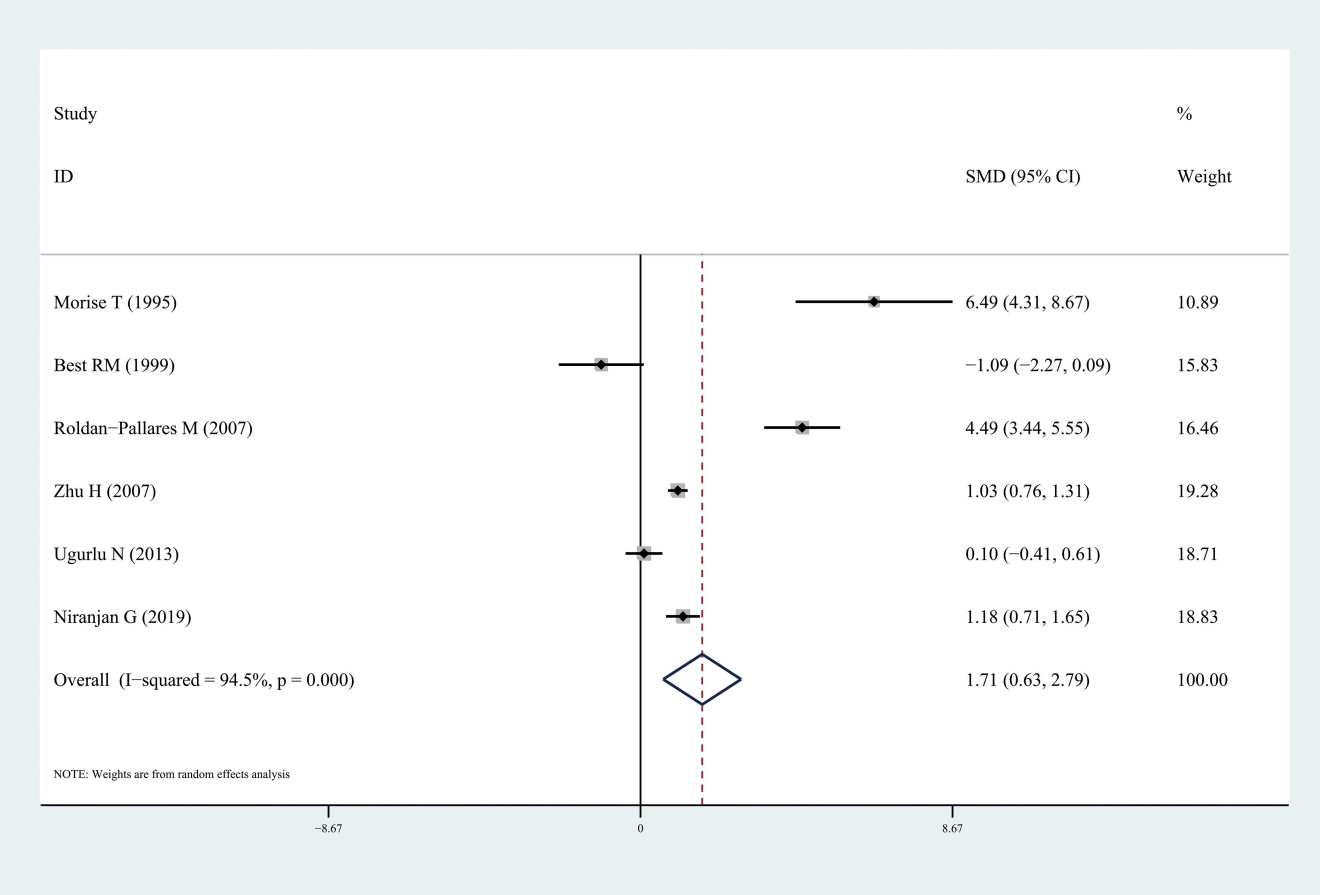
Forest plots of circulating endothelin-1 in diabetes patients with diabetic retinopathy compared to the non-diabetic retinopathy. Diamond represents the pooled SMDs at 95% CI. SMD, standardized mean difference; CI, confidence interval.

The results of this meta-analysis quantitatively showed a significant increase in circulating ET-1 levels among patients with DR (SMD: 1.73, 95% CI: 0.90, 2.56; *I^2^* = 95.2%) (**Figure 2**). In particular, ET-1 levels were markedly higher in patients with DR than in healthy individuals (SMD: 3.14, 95% CI: 1.09, 5.20; *I^2^* = 96.9%), and a significant increase was also found when comparing diabetes patients with DR to without retinopathy (SMD: 1.71, 95% CI: 0.63, 2.79; *I^2^* = 94.5%). The forest plots corresponding to these analyses were presented in **Figures 3** and **4**, respectively. One paper showed ET-1 levels were higher in patients with proliferative DR than in non-proliferative DR.

### Sensitivity analysis and publication bias

The removal of any single study in the sensitivity analysis did not substantially alter thepooled effect size, confirming the robustness of our findings (**Supplementary Figures S1**–**S3**). To assess potential publication bias, Begg’s and Egger’s statistical tests were performed following our comprehensive literature retrieval. The results from these analyses did not reveal any significant bias in the published literature included in this meta-analysis (P = 0.592 and 0.177).

## Discussion

This meta-analysis is the first to systematically evaluate ET-1 levels in patients with DR. Although previous studies have explored the association between ET-1 and DR, their findings have been inconsistent. After assessment using the NOS, all included studies are of moderate or high quality. By synthesizing data from 10 independent studies, this analysis reveals that circulating ET-1 levels are significantly elevated in patients with DR than in the control subjects (SMD: 1.73, 95% CI: 0.90, 2.56).

ET-1 is a vasoactive peptide comprising 21 amino acids. To date, four isoforms, namely, ET-1, ET-2, ET-3, and vasoactive intestinal peptide, have been identified in living organisms. These isoforms differ in gene localization, tissue expression, precursor amino acid composition, and receptor binding (11). The expression level of ET-1 is primarily regulated by chromosome 6p in endothelial cells, although it is not stored within these cells. Its release amount and rate depend on the transcriptional activity of the gene, which is influenced by the balance between activators and inhibitors. The ET-1 gene encodes a 203-amino acid precursor peptide, which is subsequently cleaved by a peptidase to form a smaller 38-amino acid peptide. Then, this intermediate is further processed by an endothelin-converting enzyme to yield mature 21-amino acid ET-1 (24). In the eye, ET-1 is most densely distributed in the choroid, followed by the iris, ciliary body, retina, optic nerve, sclera, and cornea, whereas its concentration in the vitreous humor is relatively low. ET-1 receptors are mainly located in the retina and choroid (25). However, as a peptide, it has poor corneal penetration, and it cannot be administered directly via eye drops to achieve its effects. The intravitreal injection of ET-1 in normal rabbit eyes has been shown to produce a potent and prolonged intraocular pressure-lowering effect (26). In addition, ET-1 can stimulate the proliferation and migration of corneal epithelial and endothelial cells. This promotive effect on wound healing is not accompanied with side effects such as epithelial hyperplasia, neovascularization, conjunctival hyperemia, vasoconstriction, or corneal opacity, indicating that ET-1 may act in conjunction with other factors to facilitate corneal epithelial repair (27).

The potent vasoconstrictive activity of ET-1 has prompted extensive investigation into its involvement in hypertensive disorders. Clinical relevance is underscored by the therapeutic application of endothelin receptor antagonists in pulmonary arterial hypertension (28). The vasoconstrictive and hypertensive effects of ET-1 may also provide a pathophysiological connection between elevated circulating ET-1 levels and ischemic retinopathy, particularly DR where retinal ischemia constitutes a fundamental pathological feature. Experimental studies show that intravitreal or optic nerve administration of ET-1 can induce ischemic damage characterized by retinal hypoperfusion, electroretinographic abnormalities (elevated scotopic b-wave), and ganglion cell layer apoptosis (29).

Within the retinal microenvironment, ET-1 and its Endothelin Receptor Type A (ETA) receptor have been implicated in mediating blood flow reduction during hyperglycemia and established DR. Chen et al. reported that hyperglycemia potentiates ET-1-induced vasoconstriction in human retinal venules via ETA receptor activation (30). A causative relationship between hyperglycemia and enhanced endothelial ET-1 secretion has been experimentally confirmed (31). Moreover, the level of aqueous humor ET-1 is positively correlated with DR progression, showing significant elevation in advanced stages compared with early DR and non-DR diabetic patients, which indicates that disease severity is a primary determinant of ocular ET-1 concentrations (32). Supporting evidence comes from observations of concurrent aqueous humor ET-1 elevation and impaired retinal perfusion in early nonproliferative DR (33). Collectively, ET-1 dysregulation is a potential contributor to DR pathogenesis, although aqueous humor ET-1 characterization remains relatively unexplored.

Apart from its vascular effects, ET-1 can affect metabolic homeostasis. Exogenous ET-1 administration impairs peripheral insulin sensitivity in healthy subjects (34), whereas insulin modulates vascular tone partly through ET-1 induction (35). Accumulating evidence implicating ET-1 in diabetic microangiopathy has stimulated interest in endothelin receptor antagonism as a potential DR treatment strategy. Preclinical studies provide encouraging results: Atrasentan, which is an ETA-selective antagonist, ameliorates retinal microvascular pathology in streptozotocin-induced diabetic mouse models (36). Chou et al. confirmed these protective effects, showing significant attenuation of pericyte loss following atrasentan treatment (37). Alternative administration approaches show promise, with intravitreal delivery of endothelin receptor antagonists reducing vascular leakage and downregulating VEGF and inflammatory mediators (38). Topical bosentan, which is a dual ETA/Endothelin Receptor Type B (ETB) antagonist, prevents diabetes-induced neurodegeneration in murine models through ETB receptor blockade and downregulation, providing a potential nonsystemic therapeutic route (39).

Although VEGF is a primary and well-validated target, ET-1 represents a parallel and potentially synergistic pathway, and our meta-analysis supports the rationale for further investigating its utility in patient stratification and combination therapy strategies. However, this meta-analysis, which is the first to quantitatively evaluate ET-1 levels in patients with DR, has several limitations. The overall statistical power was constrained by the predominance of small-scale studies, as large-sample case–control investigations were limited. Furthermore, significant heterogeneity was observed, which could be attributed to the variation in ET-1 detection methodologies and differences in DR severity stages across the included studies. Third, the included original studies did not provide sex-disaggregated data, we were unable to perform sex-based subgroup analyses or assess the potential moderating effect of sex on the association between ET-1 and DR. These factors may have influenced the pooled estimates, which indicates that the results should be interpreted with caution and validated in future well-designed research.

## Conclusion

This meta-analysis is the first to comprehensively evaluate the level of circulating ET-1 in patients with DR. The results of this meta-analysis show a significant association between DR and ET-1 concentration, which indicates that ET-1 is a promising biomarker for DR. Further high-quality studies are warranted to validate these findings and elucidate the underlying mechanisms.

## Data Availability

The original contributions presented in the study are included in the article/**Supplementary Material**. Further inquiries can be directed to the corresponding authors.

## Supplementary material

Supplementary Table 1 Preferred reporting items for systematic review and meta-analyses (PRISMA) checklist.

Supplementary Figure 1 The sensitivity analysis results of circulating endothelin-1 in patients with diabetic retinopathy compared to the controls.

Supplementary Figure 2 The sensitivity analysis results of circulating endothelin-1 in patients with diabetic retinopathy compared to healthy individuals.

Supplementary Figure 3 The sensitivity analysis results of circulating endothelin-1 in diabetes patients with diabetic retinopathy compared to without retinopathy.
