# Supplementary figures and images for: Association between endothelin-1 and diabetic retinopathy: a systematic review and meta-analysis
Source: Front Endocrinol (Lausanne). 2026 Mar 2;17:1754896. doi: 10.3389/fendo.2026.1754896 (PMC12989393; doi:10.3389/fendo.2026.1754896)

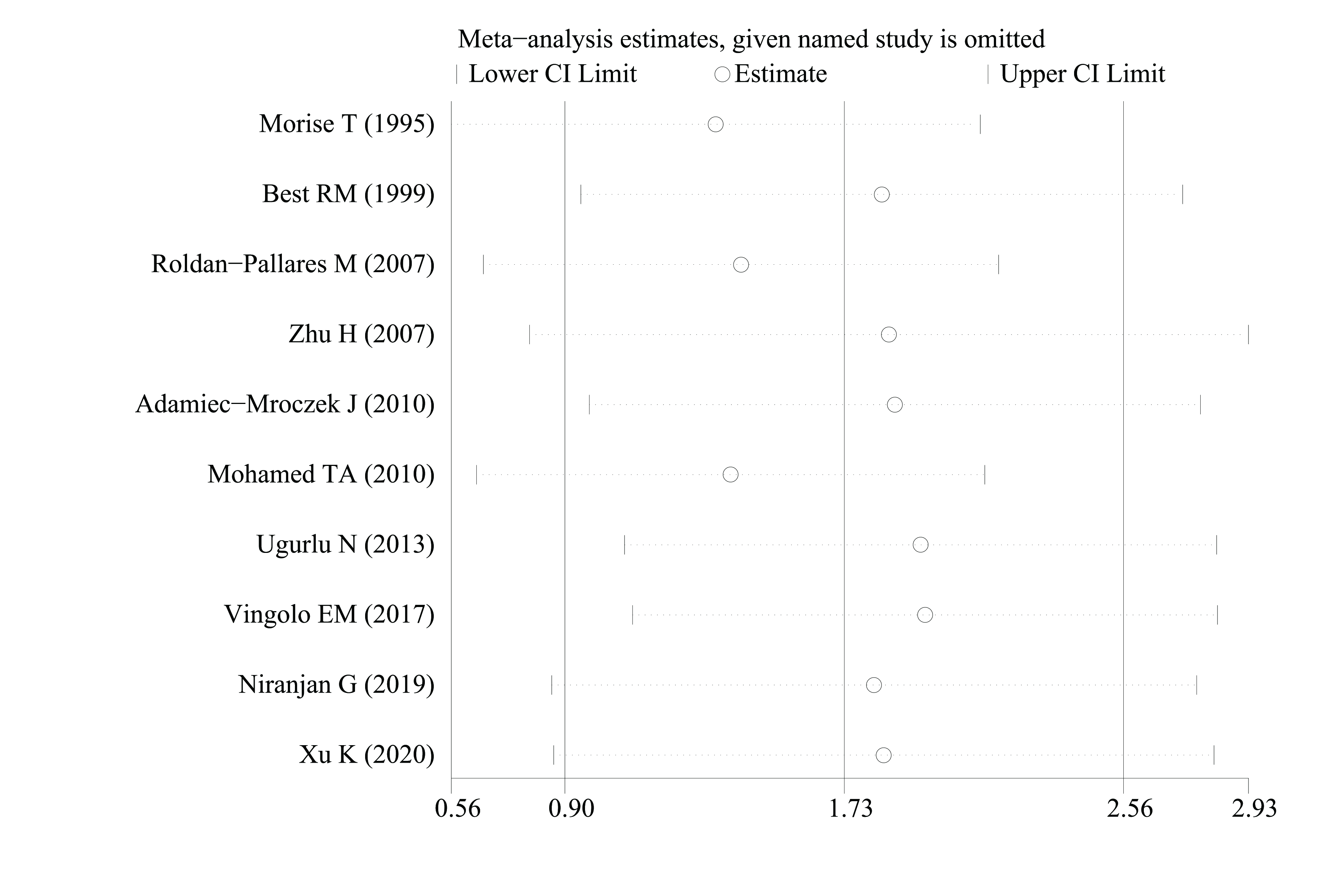

Supplement: Supplementary Figure 1 — The sensitivity analysis results of circulating endothelin-1 in patients with diabetic retinopathy compared to the controls. [file Image1.tif]

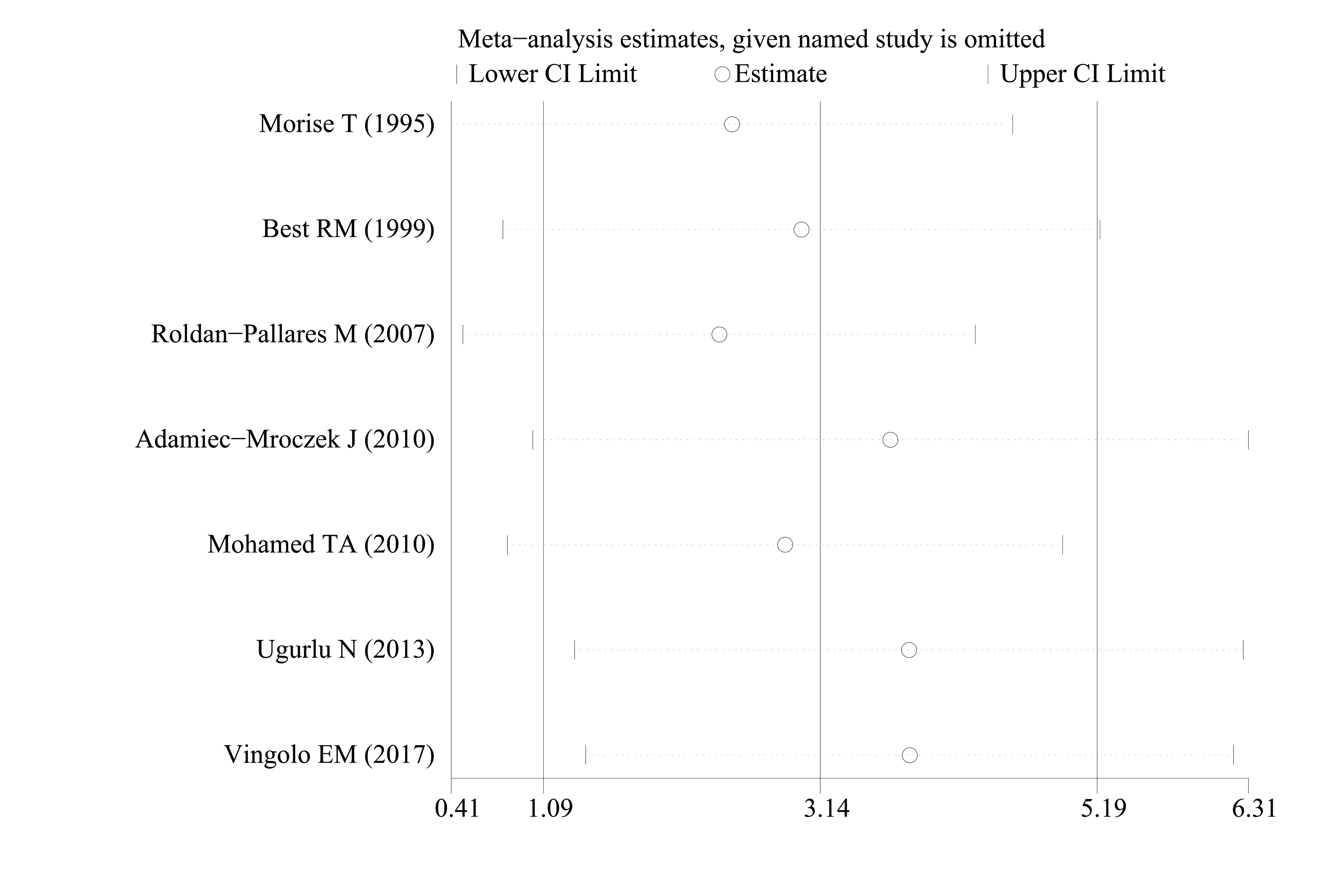

Supplement: Supplementary Figure 2 — The sensitivity analysis results of circulating endothelin-1 in patients with diabetic retinopathy compared to healthy individuals. [file Image2.tif]

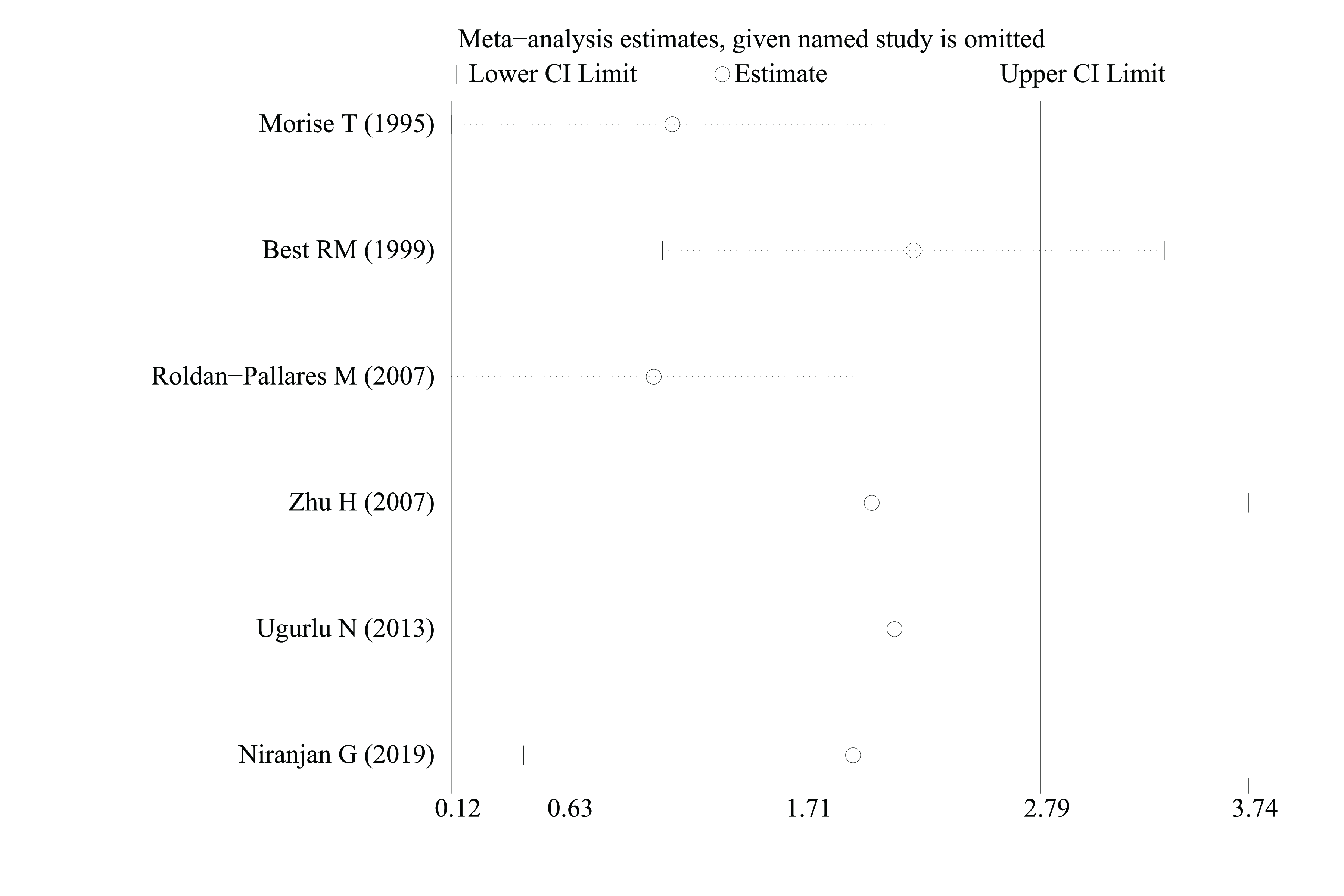

Supplement: Supplementary Figure 3 — The sensitivity analysis results of circulating endothelin-1 in diabetes patients with diabetic retinopathy compared to without retinopathy. [file Image3.tif]
